# Betulinaldehyde Ameliorates Aβ‐Induced Neurotoxicity and Cognitive Deficits by Modulating the eEF2K/eEF2 Pathway

**DOI:** 10.1002/cns.71050

**Published:** 2026-07-27

**Authors:** Chaoqun Wang, Xiaohe Han, Yali Lin, Yan Pan, Qiuping Miao, Yaping Wang, Shu‐Qin Wang, Zhu Zhang, Luyao Wang, Zhi‐Ri Tang, Yinghui Peng, Ken Kin‐Lam Yung, Nan Ma, Dan Lu, Shiqing Zhang, Lei Shi

**Affiliations:** ^1^ State Key Laboratory of Bioactive Molecules and Druggability Assessment, Guangdong Basic Research Center of Excellence for Natural Bioactive Molecules and Discovery of Innovative Drugs Jinan University Guangzhou China; ^2^ JNU‐HKUST Joint Laboratory for Neuroscience and Innovative Drug Research, College of Pharmacy Jinan University Guangzhou China; ^3^ Guangdong Province Key Laboratory of Pharmacodymamic Constituents of TCM & New Drugs Research, Guangdong Hong Kong‐Macau Joint Laboratory for Pharmacodynamic Constituents of TCM and New Drugs Research Jinan University Guangzhou China; ^4^ Department of Biology Hong Kong Baptist University Kowloon Hong Kong SAR China; ^5^ Department of Neurology and Stroke Center The First Affiliated Hospital of Jinan University Guangzhou China; ^6^ Teaching and Research Division, School of Chinese Medicine Hong Kong Baptist University Kowloon Hong Kong SAR China; ^7^ Law Sau Fai Institute for Advancing Translational Medicine in Bone & Joint Diseases (TMBJ) Hong Kong Baptist University Kowloon Hong Kong SAR China; ^8^ School of Intelligent Systems Science and Engineering Jinan University Guangzhou China; ^9^ Department of Science and Environmental Studies The Education University of Hong Kong Hong Kong Hong Kong SAR China

**Keywords:** Alzheimer's disease, Aβ_42_‐induced neurotoxicity, betulinaldehyde, eEF2K/eEF2, neuroprotection

## Abstract

**Background:**

Alzheimer's disease (AD), the most common form of dementia, remains without effective therapies. Dysregulated eukaryotic elongation factor 2 kinase/eukaryotic elongation factor 2 (eEF2K/eEF2) pathway leads to aberrant protein synthesis and impaired neuronal function in AD, positioning this axis as a promising therapeutic target. However, effective pharmacological modulators of eEF2K/eEF2 remain limited. Betulinaldehyde (Betu), an active ingredient derived from traditional Chinese medicine, remains to be thoroughly evaluated as a potential neuroprotective agent.

**Purpose:**

This study aims to investigate the therapeutic effects of Betu on AD and determine whether its activities are mediated through the eEF2K/eEF2 pathway.

**Methods:**

Neuronal cell models were used to assess the effects of Betu on β‐amyloid 42 (Aβ_42_)‐induced neuronal death, dendritic spine damage, protein synthesis inhibition, and eEF2 hyperphosphorylation. The eEF2K agonist nelfinavir (NFV) and the protein synthesis inhibitors cycloheximide (CHX) and anisomycin (AS) were used to investigate the involvement of the eEF2K/eEF2 pathway and its regulated protein synthesis in the action of Betu. The cellular thermal shift assay (CETSA) was used to detect potential interactions between Betu and eEF2K. Additionally, the effects of Betu on reactive oxygen species (ROS) levels and the associated pathways were evaluated. An Aβ_42_‐induced mouse model of AD was used to evaluate the in vivo effects of Betu on cognitive decline, hippocampal neuropathological damage, and eEF2 hyperphosphorylation.

**Results:**

Betu significantly ameliorated the Aβ_42_‐induced neuronal death and dendritic spine damage, restored protein synthesis, and reversed eEF2 hyperphosphorylation. The neuroprotective effects of Betu were effectively inhibited by the CHX, AS, or NFV treatment, and CETSA supported a potential interaction between Betu and eEF2K. Furthermore, Betu reversed Aβ_42_‐induced ROS accumulation and upregulated nuclear factor‐like 2 and heme oxygenase‐1, potential downstream effectors of the eEF2K pathway. In an Aβ_42_‐induced AD mouse model, Betu treatment markedly ameliorated cognitive decline, concomitant with attenuated neuropathological damage and restored eEF2 phosphorylation in the hippocampus.

**Conclusion:**

These findings demonstrate that Betu protects against Aβ_42_‐induced neurotoxicity by modulating the eEF2K/eEF2 pathway, highlighting its potential as a lead compound for AD.

## Graphical abstract

Betulinaldehyde exerts protective effects against Aβ_42_‐induced neuronal damage by modulating the eEF2K/eEF2 pathway to restore protein synthesis and reduce oxidative stress. In Aβ_42_‐induced mouse model, betulinaldehyde treatment alleviates hippocampal pathology and cognitive deficits, highlighting it as a potential candidate agent for AD treatment.

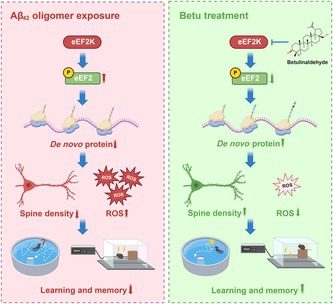

**Abbreviations**
AD
: Alzheimer's disease
ANOVA
: analysis of variance
AP
: antero‐posterior
AS
: anisomycin
Aβ
: β‐amyloid
Betu
: betulinaldehyde
CA1
: cornu ammonis 1
CETSA
: cellular thermal shift assay
CHX
: cycloheximide
DAPI
: 4′,6‐diamidino‐2‐phenylindole
DCFH‐DA
: 2′,7′‐dichlorodihydrofluorescein diacetate
DIV
: days in vitro
DMSO
: dimethyl sulfoxide
DNP
: Donepezil
DS
: Down syndrome
DV
: dorsal‐ventral
eEF2
: eukaryotic elongation factor 2
eEF2K
: eukaryotic elongation factor 2 kinase
eIF2α
: eukaryotic initiation factor 2α
eIF4B
: eukaryotic initiation factor 4B
GFAP
: glial fibrillary acidic protein
HO‐1
: heme oxygenase‐1
IBA‐1
: ionized calcium‐binding adaptor molecule‐1
IFD
: induced fit docking
MD
: molecular dynamics
ML
: medio‐lateral
mTOR
: mammalian target of Rapamycin
MTT
: 3‐(4,5‐dimethylthiazol‐2‐yl)‐2,5‐diphenyl tetrazolium bromide
MWM
: Morris water maze
NFV
: nelfinavir
NRF2
: nuclear factor‐like 2
PAH
: pulmonary arterial hypertension
PBS
: phosphate buffered saline
PD
: Parkinson's disease
p‐eEF2
: phospho‐eEF2
RMSD
: root‐mean‐square deviation
ROS
: reactive oxygen species
SEM
: standard error of the mean
SUnSET
: surface sensing of translation
**T**_**m**_
: melting temperature

## 1 Introduction

Alzheimer's disease (AD), the leading neurodegenerative condition and principal driver of dementia in the elderly population, represents a growing global health burden with prevalence anticipated to exceed 150 million cases by 2050 [1]. AD is pathologically characterized by progressive cognitive decline, synaptic dysfunction, and neuronal loss, driven primarily by extracellular β‐amyloid (Aβ) deposits and intracellular neurofibrillary tangles composed of hyperphosphorylated tau protein [2, 3]. Despite extensive research efforts spanning several decades, current treatments for AD provide mainly symptomatic benefit. The recently approved anti‐Aβ monoclonal antibodies demonstrate limited clinical efficacy, slowing the cognitive decline rather than reversing neurodegeneration [4]. This therapeutic impasse underscores the urgent need for the development of innovative therapeutic targets and intervention approaches.

Aβ oligomers impair synaptic injury, ultimately triggering neuronal death, by disrupting calcium homeostasis, overactivating glutamatergic receptors, and promoting oxidative stress. These pathways lead to impaired protein synthesis homeostasis, a fundamental requirement for synaptic plasticity and cognitive function [5, 6]. Growing evidence demonstrates that the disruption of neuronal protein synthesis serves as a key driver in AD progression, and restoring translational capacity ameliorates the cognitive impairments in preclinical models [7, 8]. Central to this regulatory mechanism is the eukaryotic elongation factor 2 kinase (eEF2K), an atypical kinase that controls the elongation phase of protein synthesis [9]. Activation of eEF2K specifically phosphorylates the Thr56 site on its sole identified substrate, eukaryotic elongation factor 2 (eEF2), and then inhibits mRNA translation [10]. Aberrant activation of eEF2K and hyperphosphorylation of eEF2 have been observed in the hippocampus of both AD patients and transgenic mouse models [11, 12], correlating with the progression of neuropathological injury. Meanwhile, eEF2K is also activated in cortical neurons induced by Aβ oligomers [13]. Several studies have suggested that genetic silencing or pharmacological inhibition of eEF2K mitigates Aβ_42_‐mediated neurotoxicity and cognitive impairments in AD mice [14, 15, 16], potentially through the restoration of protein synthesis in neurons and enhancement of nuclear factor‐like 2 (NRF2)‐mediated antioxidant response [17], indicating the essential role of the eEF2K/eEF2 pathway in Aβ‐induced neurotoxicity. Although modulating this pathway has emerged as a promising therapeutic strategy for AD, the development of effective modulators remains an unmet challenge, reinforcing the need to develop small molecule eEF2K inhibitors for AD treatment [18].

Natural products have long served as a rich source of bioactive compounds, offering chemical diversity for drug discovery. Identification of eEF2K/eEF2 modulators from natural active compounds, particularly those derived from traditional Chinese medicine, represents a promising approach for developing innovative drug candidates for AD treatment [19]. Betulinaldehyde (Betu), a triterpenoid compound found in birch bark and *Ziziphi Spinosae Semen*, exhibits a broad spectrum of biological activities, including anti‐inflammatory, anti‐oxidative, and anti‐cancer activities [20, 21, 22]. However, the neuroprotective potential of Betu in AD remains underexplored, especially regarding the modulation of aberrant protein synthesis and synaptic plasticity.

Hence, this research investigates the neuroprotective effects of Betu against Aβ‐mediated neurotoxicity and cognitive impairment, with a specific focus on its ability to modulate the eEF2K/eEF2 pathway. This study advances the understanding of Betu's therapeutic potential and mechanism in AD and demonstrates Betu as a promising natural lead compound for the eEF2K/eEF2‐targeted therapeutic strategies.

## 2 Materials and Methods

### 2.1 Animal Housing

Male adult C57BL/6 mice (8–10 weeks, 25–30 g) were purchased from Guangzhou Ruige Biological Technology Co. Ltd. These mice were accommodated in standard, specific pathogen‐free conditions. The housing environment was precisely controlled with a 12‐h light/dark cycle, 23°C ± 2°C temperature, and 55% ± 5% humidity. The mice were provided with ad libitum access to both food and water. Pregnant Sprague–Dawley rats on embryonic day 18 (E18) were obtained from the same company for primary neuronal culture. All animal procedures received approval from the Institutional Animal Care and Use Committee of Guangzhou Ruige Biotechnology (Approval No: 20230417–001).

### 2.2 Cell Culture

The mouse hippocampal neuron cell line (HT22) was purchased from Shanghai iCell Biotech Co. Ltd. (China) and maintained in culture medium supplemented with 100 U/mL of a penicillin/streptomycin mixture (Gibco, USA) and 10% fetal bovine serum (Yeasen, China) in conditions of 5% CO_2_ at 37°C. HT22 cells with passages 5 to 15 were utilized in this study. Primary hippocampal neuron cultures were prepared as established protocols [23]. Briefly, the hippocampal tissues were extracted from E18 Sprague–Dawley rat embryos into ice‐cold HBSS (Life Technologies, USA). The tissue underwent digestion in 0.05% trypsin, and the dissociated cells were placed on poly‐L‐lysine (Sigma‐Aldrich, USA) coated culture dishes in Neurobasal Medium (Gibco, USA) supplemented with 2 mM L‐Glutamine (Sigma‐Aldrich, USA), 1 mM B27 (Invitrogen, USA), and 100 U/mL penicillin/streptomycin. The primary neurons were cultured for 9 days in vitro (DIV) before experimentation.

### 2.3 Preparation of Soluble Aβ Oligomers

The recombinant β‐amyloid (1–42) peptide (A‐1163‐2, rPeptide, USA) was reconstituted to a concentration of 400 μM in 1% NH_3_·H_2_O and incubated at 37°C for 7 days to assemble Aβ_42_ oligomers. The prepared oligomers were stored at −80°C.

### 2.4 Transfection and Dendritic Spine Counting

Primary hippocampal neurons on DIV 9 were transfected with a GFP‐expressing plasmid using calcium phosphate for 12 h. On DIV 13, the transfected neurons were treated with Aβ_42_ oligomers and Betu (Purity > 99.77%, Chengdu Must Bio‐Technology Co. Ltd., China) for a duration of 24 h. The immunocytochemistry was performed on DIV 14, and the density of dendritic spines was quantified by counting the number of spines per 10 μm dendritic segment via ImageJ software (National Institute of Health, USA).

### 2.5 Cell Viability Assay

Following the cells were subjected to various treatments as specified experimental design, the solution of MTT (3‐(4,5‐dimethylthiazol‐2‐yl)‐2,5‐diphenyl tetrazolium bromide, Sigma‐Aldrich, USA) was added, and cells were further incubated at 37°C for 4 h. After incubation, the MTT solution was carefully replaced with dimethyl sulfoxide (DMSO, Sigma‐Aldrich, USA). The optical density value was monitored at 570 nm wavelength using a microplate reader (Spark, Tecan, Switzerland).

### 2.6 Surface Sensing of Translation (SUnSET) Assay

The SUnSET assay, an established technique for measuring protein synthesis in cells, was performed following our previous researches [24, 25]. The cells were treated with Betu at indicated concentrations in the presence or absence of Aβ_42_ oligomers for 1 h, including a final 30‐min incubation with or without puromycin (1 μM, Sigma‐Aldrich, USA). The puromycin‐labeled nascent proteins were detected by immunoblotting analysis with anti‐puromycin antibody, and the intensity of bands was quantified with ImageJ software.

### 2.7 Detection of Reactive Oxygen Species (ROS)

The intracellular ROS levels were detected using the fluorescent probe 2′,7′‐dichlorodihydrofluorescein diacetate (DCFH‐DA, D6883, Sigma‐Aldrich, USA). Following the treatments, HT22 cells were incubated with 15 μM DCFH‐DA in serum‐free DMEM at 37°C for 30 min. The cells were carefully rinsed and ROS levels were measured by a microplate reader at 488/525 nm. The images of cellular ROS were captured using the inverted fluorescence microscope (Axio Observer Z1, Carl Zeiss, Germany).

### 2.8 Western Blot Analysis

The protein, extracted from cells utilizing radioimmunoprecipitation assay (RIPA) buffer, was quantified using a BCA kit (Beyotime, China). Equal amounts of protein were separated by SDS‐PAGE and transferred onto polyvinylidene fluoride (PVDF) membranes (Millipore, USA). Following the transfer, the PVDF membranes were blocked and incubated with primary antibodies, including phospho‐eEF2 (Thr56) (2331S, Cell Signaling Technology, USA), eEF2 (2332S, Cell Signaling Technology, USA), eEF2K (3692S, Cell Signaling Technology, USA), Puromycin (MABE343, Millipore, USA), NRF2 (16396–1‐AP, Proteintech, USA; 12721S, Cell Signaling Technology, USA), HO‐1 (70081S, Cell Signaling Technology, USA), and β‐actin (sc‐47778, Santa Cruz Biotechnology, USA). Then, the respective secondary antibodies were incubated for 1 h at room temperature. Protein bands were captured with the Amersham Imager 600 imaging system (Cytiva, USA) and quantified using ImageJ software.

### 2.9 Molecular Dynamics (MD) Simulation

The MD simulations were performed to investigate the potential binding mode of Betu with eEF2K [26]. The structure of eEF2K (PDB ID: 8FNY) was obtained from the RCSB Protein Data Bank. The optimal docking poses of Betu were determined by using the Induced Fit Docking (IFD) protocol in Schrödinger software and selected as the starting points for subsequent MD simulations. In the Desmond module, MD simulations lasting 100 ns were performed using the OPLS4 force field. The Betu‐eEF2K complex was solvated with the TIP3P water model, and an orthogonal simulation box featuring a 10 Å buffer was constructed to eliminate boundary effects. Amounts of counterions and a 0.15 M NaCl solution were introduced to the system to neutralize the charge and simulate physiological conditions. Energy minimization of the system was conducted under the NPT ensemble (with a gradient threshold of 25 kcal/mol/Å, 300 K and 1 bar). Trajectory data were analyzed by root‐mean‐square deviation (RMSD) curves.

### 2.10 Cellular Thermal Shift Assay (CETSA)

The interaction between Betu and eEF2K in cells was analyzed using CETSA, which is based on the principle that ligand binding enhances the thermal stability of its target protein. The HT22 cells were lysed in PBS containing phosphatase and protease inhibitors, followed by repeated freeze–thaw cycles in liquid nitrogen to achieve cytolysis. After centrifugation for 20 min (17,000 × g, 4°C), the supernatant was incubated with DMSO or Betu (80 μM) at 37°C for 1 h. The samples were then divided into 7 aliquots and heated for 3 min at 51°C, 55°C, 59°C, 63°C, 67°C, 71°C, and 75°C, respectively. Following heat treatment, samples were centrifuged and the supernatant was analyzed by Western blot.

### 2.11 Aβ_42_‐Induced AD Mouse Model and Treatment

The mice were anesthetized with 1.25% tribromoethanol via intraperitoneal injection (i.p.) and secured in a stereotaxic frame (RWD Life Science, China). Aβ_42_ oligomers solution (1 μg/μl, 2 μL) or vehicle was bilaterally injected (0.3 μL/min) into the hippocampal cornu ammonis 1 (CA1) region using stereotaxic coordinates: antero‐posterior (AP): −2.33 mm, medio‐lateral (ML): ±1.8 mm, dorsal‐ventral (DV): −1.5 mm. The needle was gradually retracted after 8 min of stationary placement. For treatment, Betu or Donepezil (DNP, Sigma‐Aldrich, USA) was dissolved in saline containing 2% DMSO, 5% TWEEN (Sigma‐Aldrich, USA) and 30% PEG‐400 (MedChemExpress, USA). Mice were daily administered with vehicle, Betu (25, 100 mg/kg), or DNP (5 mg/kg) via oral gavage for 7 days prior and 21 days post Aβ_42_ injection. The behavior tests were performed, and drug treatment was continued until the tests were completed. Mice were then anesthetized with 2% isoflurane. Blood was harvested from the heart for serum biochemistry analysis, and tissues were collected for pathological analysis (Figure S1).

### 2.12 Morris Water Maze (MWM) Test

The procedure of the MWM test was followed by the previous study [27]. The apparatus consisted of a circular tub (100 cm in diameter) filled with water (15 cm in height, at 23°C), added with skim milk powder. In the training phase, an escape platform was placed in a fixed quadrant. Mouse was allowed 60 s to locate the platform. Once failed, the mouse was guided to the platform and allowed to remain for 15 s. The training phase was repeated for five consecutive days. The probe trial was conducted 24 h after the last training trial, allowing each mouse to swim freely for 60 s with the platform removed. The movements of the mice were recorded.

### 2.13 Step‐Down Avoidance Test

The long‐term aversive memory was assessed by the step‐down avoidance test as previously described [28]. During the training phase, mice were allowed a 5 min adaptation period on the grid floor, followed by an electric shock (45 V, 0.25 mA, 1 Hz) for 5 min with 2‐s intervals. Mice that failed to jump to the platform were excluded. The testing phase was conducted 24 h later for 7 consecutive days, with no shocks being administered to the grid floor. The latency to step down within 5 min and the number of step‐down events of each mouse were recorded.

### 2.14 Golgi Staining

Neuronal morphology and spine density of neurons were assessed by a Golgi‐Cox staining kit (G1069, Servicebio, China). Brain samples were immersed in Golgi‐Cox solution away from the light at room temperature, and then the tissue was sliced into 60 μm‐thick sections. After washing, sections were treated with ammonia solution and mounted. Images were obtained using a slide scanner (Pannoramic MIDI, 3DHISTECH Ltd., Hungary), and spine density (spines per 10 μm) was quantified with ImageJ software.

### 2.15 Immunofluorescence

The immunofluorescence was performed as described previously [29]. Coronal brain sections were blocked for 1 h at room temperature, incubated with primary antibodies overnight at 4°C, followed with secondary antibodies for 1 h, and then incubation of 4′,6‐diamidino‐2‐phenylindole (DAPI, 62248, Invitrogen, USA) for 10 min at room temperature. Antibodies: anti‐GFAP (GB12096, Servicebio, China), anti‐IBA1 (GB12105‐100, Servicebio, China), anti‐p‐eEF2 (Thr56) (2311 s, Cell signaling technology, USA), alexa fluor 546 goat anti‐rabbit immunoglobulin G (IgG) (A11035, Invitrogen, USA), and alexa fluor 488 goat anti‐mouse IgG (A11029, Invitrogen, USA). Images were obtained using a digital slice scanner and confocal microscopy (LSM 800, Carl Zeiss, Germany). The number of GFAP^+^ and IBA‐1^+^ cells in the hippocampal CA1 regions and the signal density of phospho‐eEF2 (p‐eEF2) were analyzed using ImageJ software.

### 2.16 Statistical Analysis

Data were presented as mean ± standard error of the mean (SEM) from at least 3 independent experiments. The statistical analyses were performed with GraphPad Prism 9.5 software and significance was assigned at *p* < 0.05. Multiple group comparisons were performed using either one‐way or two‐way ANOVA with Dunnett's post hoc test, while nonparametric datasets were evaluated using the Kruskal‐Wallis test.

## 3 Results

### 3.1 Betu Protects Hippocampal HT22 Cells Against Aβ_42_‐Induced Neurotoxicity by Enhancing Protein Synthesis

**FIGURE 1:**
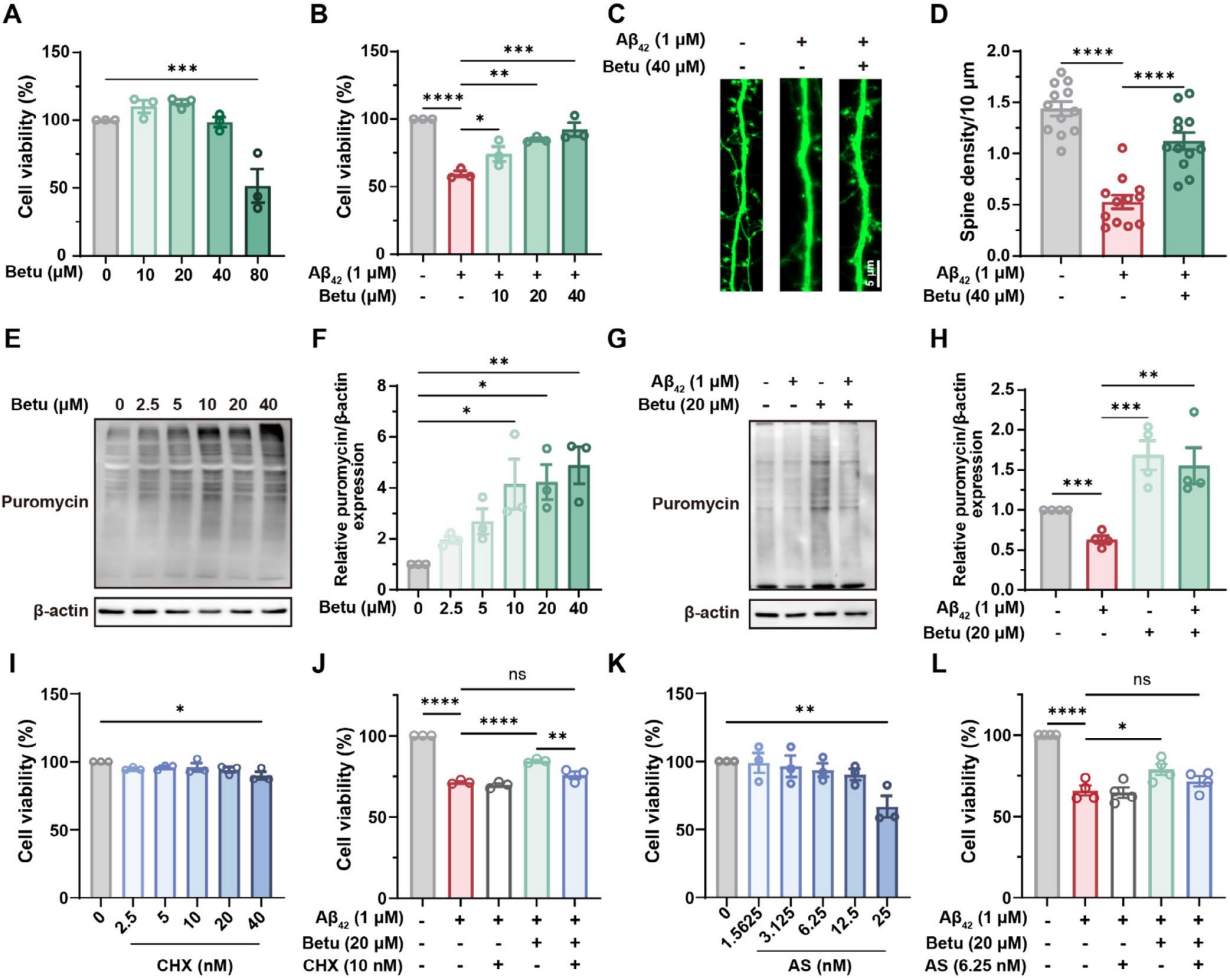
Betu mitigates Aβ_42_‐induced toxicity by enhancing protein synthesis. (A) The effect of Betu (0, 10, 20, 40, 80 μM) on HT22 cell viability for 24 h exposure (*n* = 3). (B) Dose‐dependent effect of Betu (10, 20, 40 μM) against Aβ_42_‐mediated cell toxicity in HT22 cells (*n* = 3). (C) Representative fluorescence images of dendritic spines in primary hippocampal neurons after co‐treatment with Aβ_42_ and Betu (40 μM) for 24 h. Scale bar: 5 μm. (D) Quantification of spine density/10 μm (*n* = 12 cells from 3 independent experiments). (E) Representative Western blot images of puromycin expression in HT22 cells treated with Betu (0, 2.5, 5, 10, 20, 40 μM) for 1 h. (F) Quantitative analysis of puromycin/β‐actin (*n* = 3). (G) Representative Western blot images of puromycin expression in HT22 cells after co‐treatment with Aβ_42_ (1 μM) and Betu (20 μM) for 1 h. (H) Quantitative analysis of puromycin/β‐actin (*n* = 4). (I) The effect of CHX (2.5, 5, 10, 20, 40 nM) on HT22 cell viability for 24 h (*n* = 3). (J) The effect of co‐treatment with Aβ_42_ (1 μM), Betu (20 μM), and CHX (10 nM) on HT22 cell viability for 24 h (*n* = 3). (K) The effect of AS (1.5625, 3.125, 6.25, 12.5, 25 nM) on HT22 cell viability for 24 h (*n* = 3). (L) The effect of co‐treatment with Aβ_42_ (1 μM), Betu (20 μM), and AS (6.25 nM) on HT22 cell viability for 24 h (*n* = 4). *p < 0.05, **p < 0.01, ***p < 0.001, ****p < 0.0001. One‐way ANOVA followed by Dunnett's multiple comparisons test. Data are presented as mean ± SEM.

To determine the noncytotoxic dosage range, HT22 cells were treated with Betu at different concentrations (10, 20, 40, and 80 μM) for 24 h. Cell viability assays demonstrated that Betu exhibited no significant cytotoxicity at concentrations up to 40 μM (Figure 1A). Subsequently, the protective effect of Betu was assessed by co‐treating HT22 cells with Aβ_42_ oligomers (1 μM) and Betu (10, 20, and 40 μM). The results revealed that Aβ_42_ exposure significantly reduced cell survival by approximately 50% compared to the control group, while Betu significantly restored cell viability in a dose‐dependent manner (Figure 1B). In primary hippocampal neurons, Aβ_42_ exposure markedly reduced dendritic spine density, and this damage was significantly ameliorated by treatment with Betu (40 μM) (Figure 1C,D). Given the central role of protein synthesis in maintaining neuronal function and synaptic plasticity [30], we monitored the effect of Betu on *de novo* protein synthesis by the SUnSET assay. The results showed that Betu significantly and dose‐dependently increased neural protein synthesis (Figure 1E,F). Furthermore, Aβ_42_ oligomers significantly inhibited protein synthesis, which was effectively reversed by Betu treatment (Figure 1G,H). To further validate whether Betu‐induced protein synthesis enhancement is required for its neuroprotective effect against Aβ_42_‐induced toxicity, the protein synthesis inhibitors cycloheximide (CHX) [31] and anisomycin (AS) [32] were applied to investigate their impact on the protective effects of Betu. The CHX (10 nM) or AS (6.25 nM) did not affect cell viability alone, but they both significantly abolished the protective effects of Betu against Aβ_42_‐induced neurotoxicity (Figure 1I–L). These results collectively indicate that the neuroprotective effects of Betu depend primarily on its ability to enhance protein synthesis.

### 3.2 Betu Alleviates Aβ_42_‐Induced Neurotoxicity via Regulation of eEF2K/eEF2 Pathway

**FIGURE 2:**
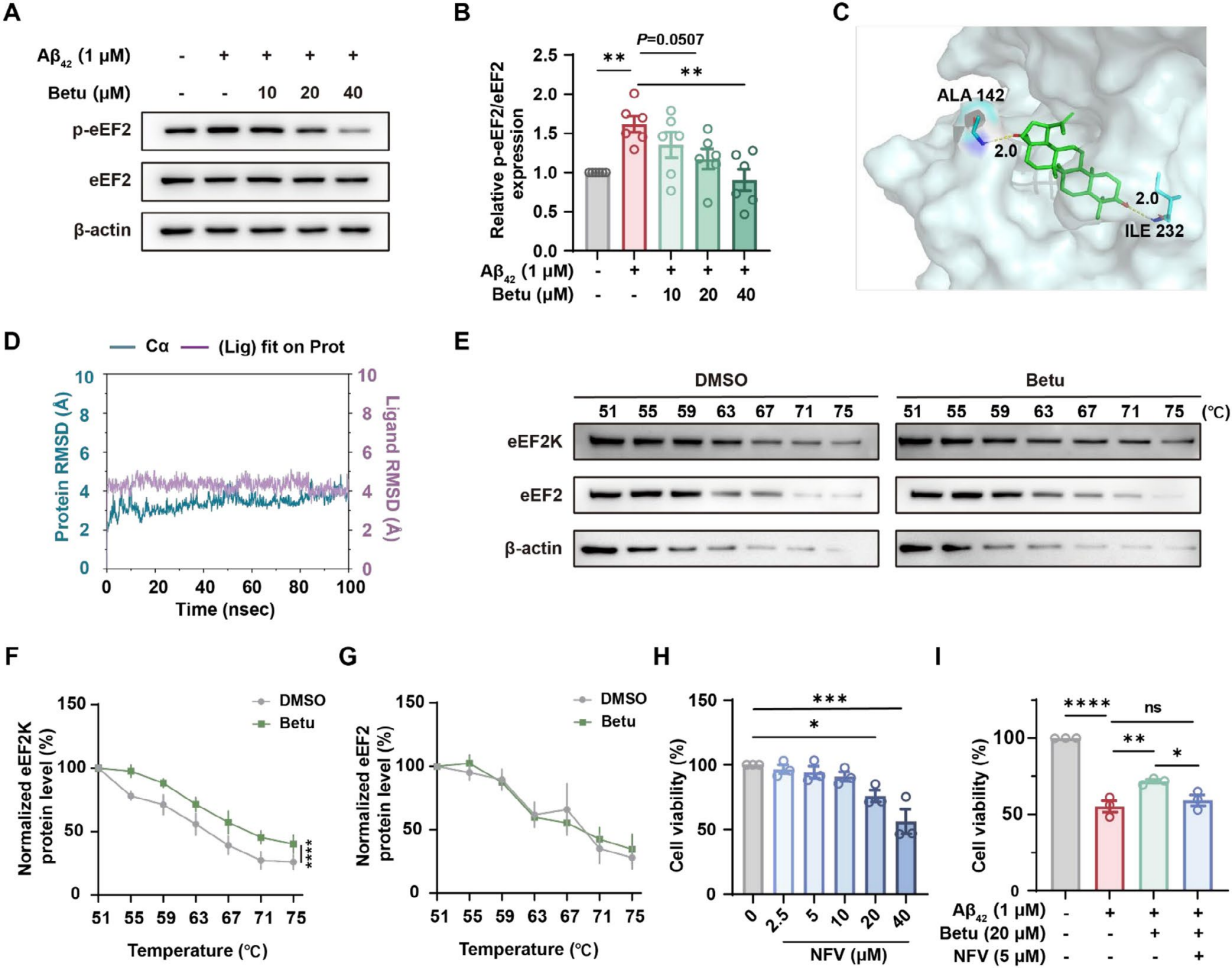
Betu alleviates Aβ_42_ toxicity via regulation of the eEF2K/eEF2 pathway. (A) Representative Western blot images of p‐eEF2 and eEF2 expression in HT22 cells after co‐treatment with Aβ_42_ (1 μM) and Betu (10, 20, 40 μM) for 24 h. (B) Quantitative analysis of p‐eEF2/eEF2 (*n* = 6). **p < 0.01. One‐way ANOVA followed by Dunnett's multiple comparisons test. (C) Induced fit docking (IFD) pose of the Betu docked with the eEF2K protein. (D) The root‐mean‐square deviation (RMSD) plot from molecular dynamics (MD) simulation of Betu–eEF2K complex. (E) Representative Western blot images of the changes in thermal stability of eEF2K, eEF2, and β‐actin in HT22 cell lysate treated with DMSO or Betu. (F–G) Cellular thermal shift assay (CETSA) curves of eEF2K (F) and eEF2 (G) were determined in the absence and presence of Betu, normalized to signal at 51°C (*n* = 4). ****p < 0.0001. Two‐way ANOVA test. (H) The effect of NFV (2.5, 5, 10, 20, 40 μM) on HT22 cell viability for 24 h (*n* = 3). *p < 0.05, ***p < 0.001. One‐way ANOVA followed by Dunnett's multiple comparisons test. (I) The effect of co‐treatment with Aβ_42_ (1 μM), Betu (20 μM), and NFV (5 μM) on HT22 cell viability (*n* = 3). *p < 0.05, **p < 0.01, ****p < 0.0001. One‐way ANOVA followed by Dunnett's multiple comparisons test. Data are presented as mean ± SEM.

To investigate the mechanism of Betu on promoting neuronal protein synthesis, we monitored the expression of translation factors in initiation stage (eukaryotic initiation factor 2α, eIF2α; eukaryotic initiation factor 4B, eIF4B) and elongation stage (eEF2). After the HT22 cells were treated with Aβ_42_ (1 μM) in the presence of Betu (10, 20, and 40 μM) for 24 h, Western blot analysis revealed that Betu did not alter the phosphorylation level of eIF2α and eIF4B (Figure S1). However, Aβ_42_ exposure significantly elevated the phosphorylation level of eEF2 compared to the control cells, which was significantly reversed by Betu treatment in a dose‐dependent manner (Figure 2A,B), suggesting that Betu promoted protein synthesis by modulating eEF2 phosphorylation. Given that eEF2 is phosphorylated at Thr56 by its sole upstream kinase eEF2K and then inhibits the process of protein synthesis [33], we further examined whether Betu binds to and inhibits eEF2K activity. Computational studies including IFD and MD simulations were conducted to investigate potential interactions between Betu and eEF2K. Amino acid residues ALA142 and ILE232 of eEF2K contributed to the binding affinity (Figure 2C). Notably, both ALA142 and ILE232 were reported to be involved in the binding of eEF2K with ATP and its selective inhibitor A‐484954, suggesting that Betu potentially blocks the eEF2K activity by competitively inhibiting ATP binding [26, 34, 35]. In addition, the RMSD plot revealed the stability of Betu‐eEF2K complex during the process of 100 ns MD simulation (Figure 2D). To verify whether Betu directly binds to eEF2K or eEF2, a CETSA assay was performed. The results showed that Betu increased the thermal stability of eEF2K compared to the control group, while the stability of eEF2 and β‐actin remained unchanged (Figure 2E–G and Figure S2). By plotting the relative levels of eEF2K protein against temperatures, thermal shift curves were generated and the melting temperatures (T_m_) were calculated. The mean T_m_ value of eEF2K rose from 64.71°C in the control group to 70.27°C in the Betu‐treated group, indicating that Betu directly binds to and stabilizes eEF2K (Figure 2F). Functionally, the eEF2K activator nelfinavir (NFV) [36] was utilized to assess its effect on Betu's protective actions. The results demonstrated that NFV (5 μM) significantly attenuated the protective effects of Betu against Aβ_42_‐induced cytotoxicity (Figure 2H,I). Collectively, the above studies indicate that Betu promotes protein synthesis and neuroprotection predominantly by inhibiting eEF2K and then reducing eEF2 phosphorylation.

### 3.3 Betu Ameliorates Aβ_42_‐Induced ROS Production

**FIGURE 3:**
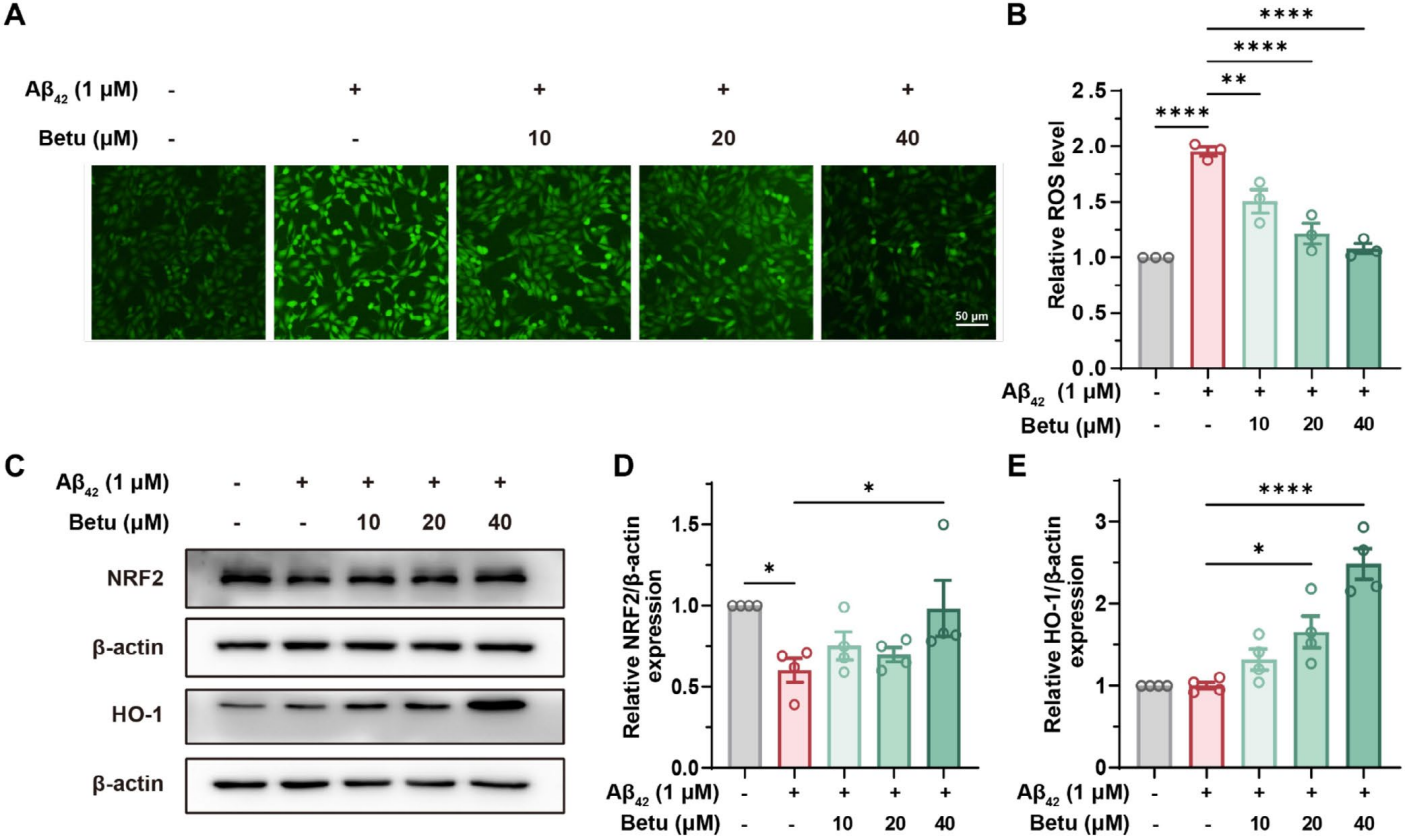
Betu ameliorates Aβ_42_‐induced ROS overproduction and activates NRF2/HO‐1 pathway. (A) Representative fluorescence images of ROS in HT22 cells after co‐treatment of Aβ_42_ (1 μM) and Betu (10, 20, 40 μM) for 24 h. Scale bar: 50 μm. (B) Quantitative results of ROS fluorescence intensity in HT22 cells (*n* = 3). (C) Representative Western blot images of NRF2 and HO‐1 expression in HT22 cells after co‐treatment with Aβ_42_ and Betu for 24 h. (D, E) Quantitative analysis of NRF2/β‐actin (D) and HO‐1/β‐actin (E) (*n* = 4). *p < 0.05, **p < 0.01, ****p < 0.0001. One‐way ANOVA followed by Dunnett's multiple comparisons test. Data are presented as mean ± SEM.

Aβ_42_‐associated oxidative stress is a critical contributor to its neurotoxicity and cognitive impairment in the pathology of AD. Researchers also indicated that protein synthesis and the eEF2K pathway play a crucial role in regulating cellular oxidative stress damage [37, 38]. In this study, the effect of Betu on Aβ_42_‐induced oxidative stress was investigated. Our findings demonstrated a significant increase in ROS production in HT22 cells after Aβ_42_ exposure, while Betu treatment at concentrations of 10, 20, and 40 μM significantly and dose‐dependently decreased the ROS production (Figure 3A,B). Considering that eEF2K inhibition can enhance the NRF2‐mediated antioxidant response in neurons [17], the activation of the NRF2/HO‐1 pathway was monitored after Aβ_42_ exposure and Betu treatment. Consistently, the results observed that Aβ_42_ induction significantly reduced the level of NRF2 in HT22 cells without altering HO‐1 levels. In contrast, Betu administration potently enhanced the expression of both NRF2 and HO‐1 proteins (Figure 3C–E). These results suggest that Betu alleviates the overproduction of ROS induced by Aβ_42_ by enhancing the NRF2‐mediated antioxidant response, which is one of the downstream effects of eEF2K inhibition [17].

### 3.4 Betu Ameliorates the Learning and Memory Decline in Aβ_42_‐Modeled Mice

**FIGURE 4:**
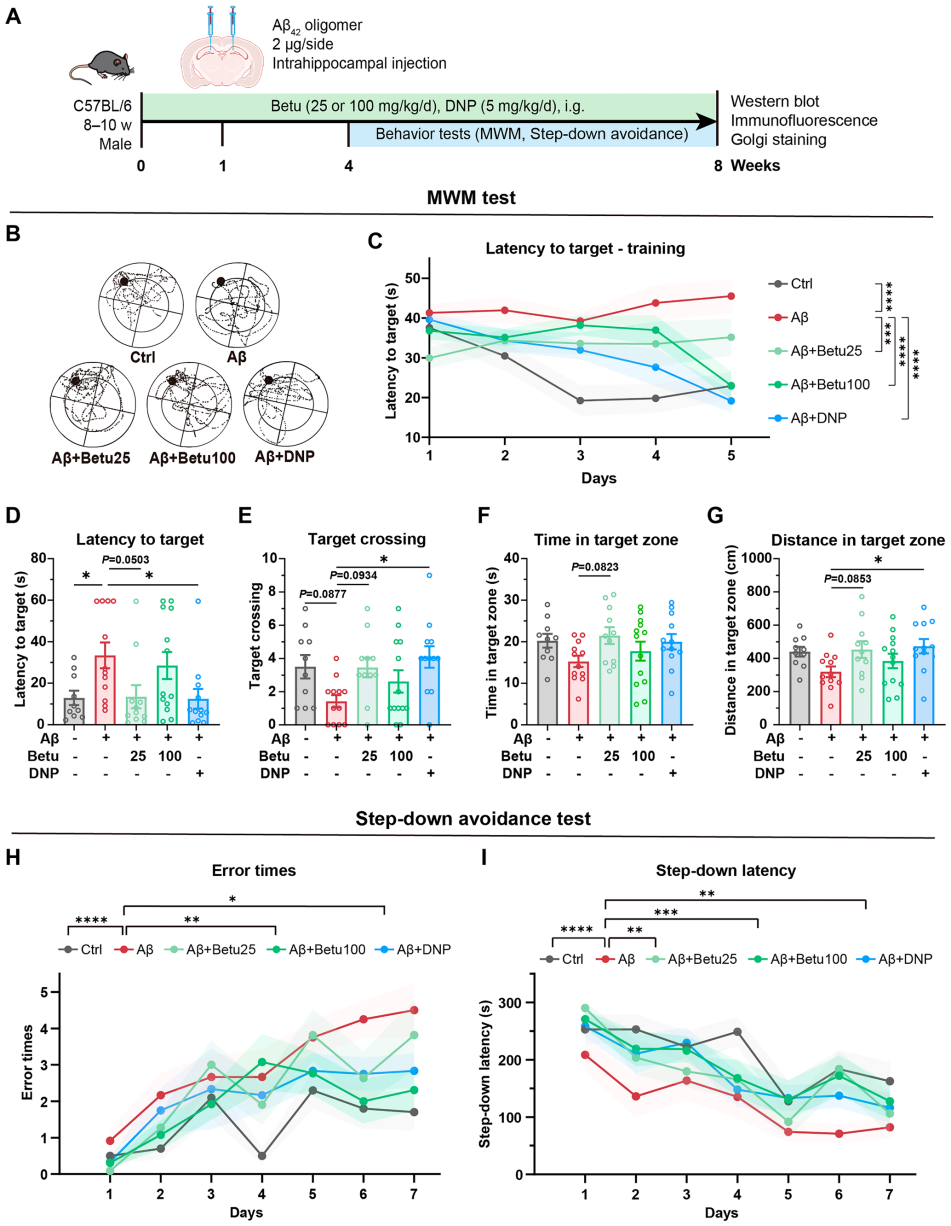
Betu administration alleviates cognitive decline in Aβ_42_‐modeled mice. (A) Schematic representation of the experimental timeline of drug administration and behavioral testing. The Morris water maze (MWM) (B–G) and step‐down avoidance (H, I) tests were conducted to assess the cognitive function in mice. (B) Representative swim path of MWM test. (C) The latency to target over five consecutive training days in the MWM test. ***p < 0.001, ****p < 0.0001. Two‐way ANOVA followed by Dunnett's multiple comparisons test. (D–G) The latency to target (D), number of target crossings (E), time in target zone (F), and distance in target zone (G) during the probe test trial in the MWM test. *p < 0.05. One‐way ANOVA followed by Dunnett's multiple comparisons test. (H, I) The error times (H) and step‐down latency (I) in the step‐down avoidance test. *p < 0.05, **p < 0.01, ***p < 0.001, ****p < 0.0001. Two‐way ANOVA followed by Dunnett's multiple comparisons test. Group sizes: Ctrl (*n* = 10), Aβ + model (*n* = 12), Aβ + Betu‐25 (*n* = 11), Aβ + Betu‐100 (*n* = 13), Aβ + DNP (*n* = 12). Data are presented as mean ± SEM.

To evaluate the efficacy of Betu in vivo, an AD model was established by intrahippocampal injection of Aβ_42_ and the dosage used was dependent on a previous study [20]. The mice were orally administered Betu (25 or 100 mg/kg), DNP (5 mg/kg), or vehicle for 7 days before and 21 days after Aβ_42_ injection, and the cognitive function of mice after administration was then evaluated using the MWM and step‐down avoidance tests (Figure 4A). In the MWM test, Aβ_42_‐modeled mice exhibited a significantly increased latency to locate the platform in the training session compared to the control mice. Notably, mice treated with Betu (25 and 100 mg/kg) or DNP exhibited markedly shorter latency compared with the Aβ_42_‐modeled group. (Figure 4B,C). During the probe test, Aβ_42_‐modeled mice showed increased latency, decreased target crossing times, and reduced time and distance traveled in the target zone, indicating the impaired spatial memory after Aβ_42_‐injection. However, Betu treatment reduced the escape latency, increased target crossings, and improved the time and distance traveled in the target zone compared to the model mice (Figure 4D–G). In addition, the step‐down avoidance test was utilized to assess the aversive memory. Over a seven‐day trial period, Aβ_42_‐modeled mice demonstrated significant memory impairment, characterized by increased error times and reduced latency to step down from the platform compared to the control mice; however, these deficits were significantly ameliorated by the treatment with Betu in a dose‐dependent manner (Figure 4H,I). Thus, the behavioral assessments indicate that Betu significantly ameliorates the learning and memory impairments in Aβ_42_‐modeled mice.

### 3.5 Betu Attenuates Neuropathological Damages and eEF2 Hyperphosphorylation in the Hippocampus of Aβ_42_‐Modeled Mice

**FIGURE 5:**
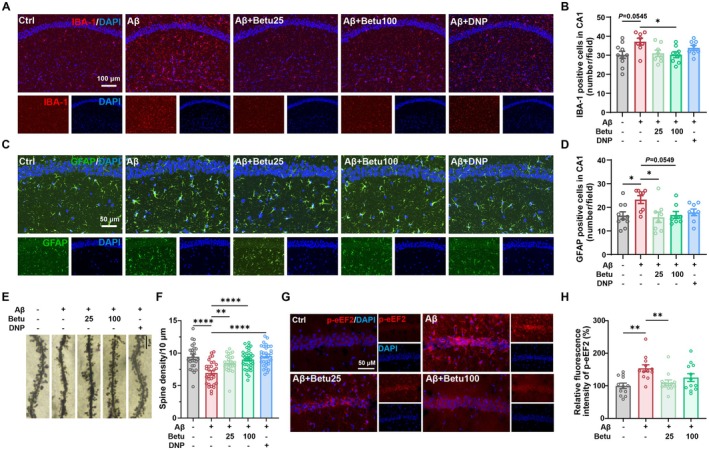
Betu mitigates neurogliosis, spine loss, and hyperphosphorylation of eEF2 in Aβ_42_‐modeled mice. (A) Representative fluorescence images of IBA‐1^+^ cells in the hippocampal CA1 region. Red: IBA‐1, blue: 4′,6‐diamidino‐2‐phenylindole (DAPI). Scale bar: 100 μm. (B) Quantification of IBA‐1^+^ cell density, *n* = 8–10 regions from 4 to 5 mice. *p < 0.05. Kruskal‐Wallis test followed by Dunn's multiple comparison test. (C) Representative fluorescence images of GFAP^+^ cells in the hippocampal CA1 region. Green: GFAP, blue: DAPI. Scale bar: 50 μm. (D) Quantification of GFAP^+^ cell density, *n* = 8–10 regions from 4 to 5 mice. *p < 0.05. Kruskal‐Wallis test followed by Dunn's multiple comparison test. (E) Representative image of Golgi‐stained dendritic spine in the hippocampal CA1 region. Scale bar: 5 μm. (F) Quantification of dendritic spine density, *n* = 25–35 cells from 5 to 7 mice. **p < 0.01, ****p < 0.0001. One‐way ANOVA followed by Dunnett's multiple comparisons test. (G) Representative immunofluorescence images of p‐eEF2 in the hippocampal CA1 region. Red: P‐eEF2, blue: DAPI. Scale bar: 50 μm. (H) Quantification of immunofluorescence intensity of p‐eEF2, *n* = 12 regions from 6 mice. **p < 0.01. One‐way ANOVA followed by Dunnett's multiple comparisons test. Data are presented as mean ± SEM.

The reactive gliosis of microglia (IBA‐1) and astrocytes (GFAP) in the hippocampus was monitored using immunofluorescent staining. Aβ_42_‐modeled mice exhibited a remarkable increase in IBA‐1^+^ cells in the hippocampal CA1 region. Administration of Betu and DNP reduced microglial activation, with a significant difference observed in the 100 mg/kg Betu group (Figure 5A,B). Similarly, the number of GFAP^+^ astrocytes was reduced in Aβ_42_‐modeled mice after Betu or DNP treatment, with a significant difference noted in the 25 mg/kg Betu group (Figure 5C,D). Furthermore, Golgi staining was performed to evaluate the morphology and density of dendritic spines in the hippocampal CA1 region. The results revealed that Aβ_42_ reduced dendritic spine density and shifted spine morphology from mushroom to thin, filamentous spines. However, treatment with Betu or DNP significantly counteracted the Aβ_42_‐induced dendritic spine damage (Figure 5E,F). These findings are consistent with our in vitro results, which showed that Betu significantly mitigated Aβ_42_‐induced dendritic spine loss in primary neurons (Figure 1C,D). We next investigated whether the phosphorylation level of eEF2 in the hippocampus is affected by Betu treatment. As expected, immunofluorescence revealed that Aβ_42_ injection significantly increased the eEF2 phosphorylation in the hippocampal CA1 region of mice, and this effect was substantially ameliorated by Betu treatment, with a significant difference in the 25 mg/kg group (Figure 5G,H). To evaluate the potential toxicity of Betu, the body weight and major tissue pathology were monitored and no significant alterations were detected (Figure S3). Meanwhile, several serum biochemical markers were detected, including alanine aminotransferase (ALT), aspartate aminotransferase (AST), creatinine (CREA), uric acid (UA), triglycerides (TG), and total cholesterol (CHO). Betu treatment did not markedly affect most of these markers compared to the model group, except for a significant reduction in UA at high dosage (Figure S3). Collectively, these findings suggest that Betu alleviates Aβ_42_‐induced neuropathological damages and eEF2 hyperphosphorylation, in parallel with improved cognition in mice, supporting eEF2K/eEF2 inhibition as a main mechanism underlying the neuroprotective effect of Betu.

## 4 Discussion

AD represents a significant global health challenge, neuropathologically characterized by extracellular Aβ plaques, underscoring the urgent need for novel therapeutic targets and drug candidates. Aβ accumulation has long been implicated in synaptic dysfunction and neuronal loss. Emerging evidence indicates that disruption of protein homeostasis, a fundamental process essential for maintaining synaptic plasticity, plays a critical role in the neuropathological damage of AD [39]. Protein synthesis occurs in three ordered steps: initiation, elongation, and termination [40]. Multiple interconnected pathways may mediate Aβ‐induced protein synthesis deficits. Chronic endoplasmic reticulum stress, triggered by Aβ oligomers, activates the unfolded protein response, leading to sustained phosphorylation of eIF2α [41, 42]. Notably, phosphorylated eIF2α is elevated in AD models and postmortem brains, correlating with reduced levels of plasticity‐related proteins [7]. Concurrently, Aβ may dysregulate the mammalian target of rapamycin (mTOR) signaling, the central regulator of cap‐dependent translation. Aβ aggregates suppress mTORC1, which normally phosphorylates and inhibits eEF2K via ribosomal protein S6 kinase (S6K) [43]. When Aβ impairs mTORC1 activity, eEF2K is no longer adequately suppressed, leading to increased eEF2 phosphorylation. Indeed, abnormal hyperphosphorylation of eEF2 has been documented in AD models and postmortem brain tissues, which inhibits the elongation step of protein synthesis and contributes to neural damage and cognitive impairments [33].

Among these regulatory pathways, recent advances have highlighted the eEF2K/eEF2 pathway as a critical contributor to the Aβ pathology and AD progression. Importantly, inhibiting the activity of eEF2K ameliorates synaptic deficits and cognitive impairments in AD mouse models [11, 44]. The eEF2K/eEF2 pathway has emerged as a promising therapeutic target for AD, with a potentially favorable safety profile given that eEF2K is not essential for mammalian development or cell viability [45]. Additionally, as an atypical kinase, eEF2K is unlikely to broadly affect other protein kinases, minimizing the off‐target effects [46]. The eEF2K/eEF2 pathway represents a distinct therapeutic strategy compared to conventional Aβ‐targeted approaches. The recently developed anti‐Aβ immunotherapies primarily promote plaque clearance but have limited efficacy in reversing synaptic loss in AD [47]. A therapeutic strategy based on eEF2K directly counteracts downstream consequences of Aβ‐mediated toxicity and may offer neuroprotection at advanced disease stages. However, no anti‐AD drugs targeting the eEF2K/eEF2 pathway have been marketed or reached clinical trials, emphasizing the need for further research [48].

The present study underscores the significance of Betu, a natural triterpenoid compound, in addressing the Aβ_42_‐mediated neurotoxicity through the modulation of the eEF2K/eEF2 pathway, thereby offering a potential therapeutic strategy for AD. Specifically, Betu effectively prevented neuronal death, dendritic spine damage, protein synthesis inhibition, and eEF2 hyperphosphorylation induced by Aβ_42_. The MD simulation and CETSA support direct binding of Betu to eEF2K. Furthermore, the protein synthesis inhibitors (CHX, AS) or eEF2K agonist (NFV) effectively attenuated Betu's efficacy, underscoring the crucial role of protein synthesis and the eEF2K/eEF2 pathway in its mechanism of action. In vivo, Betu improved spatial and fear memory and restored dendritic spine density, and these cognitive benefits are likely mediated by suppression of eEF2 phosphorylation in the hippocampal CA1 region of Aβ_42_‐modeled mice. Additionally, Aβ aggregation has been implicated in mitochondrial dysfunction, leading to elevated ROS levels. Our results indicate that the antioxidant effects of Betu may involve the activation of the NRF2/HO‐1 pathway, which has been reported as a downstream effector of the eEF2K pathway [17]. Although the mechanistic link between eEF2K inhibition and NRF2/HO‐1 activation requires further elucidation, our findings provide a rationale for further investigation. Furthermore, elevated UA and CHO have been reported to be implicated in AD progression [49, 50]. Our study also showed that Aβ_42_ increased serum levels of UA and CHO in mice, which were reduced by Betu treatment, suggesting additional benefits of Betu on AD‐related metabolic dysregulation.

In addition to AD, hyperactivation of eEF2K represents a crucial pathogenic mechanism in several other neurological diseases, including Parkinson's disease (PD) and Down syndrome (DS). For example, eEF2K activity is elevated in PD brains, and eEF2K suppression protects against α‐synuclein toxicity [51]. eEF2K signaling is also hyperactivated in the brains of patients and mouse models of DS. Suppression of eEF2K and then eEF2 dephosphorylation improves protein synthesis deficiency and synaptic morphological defects in the DS mouse model [52]. Beyond neurological diseases, eEF2K is a prospective therapeutic target in oncology and cardiovascular diseases [53]. It has been revealed that excessive eEF2K expression correlates with poor prognosis in lung cancer patients, supporting eEF2K inhibition as a novel therapeutic avenue [54]; notably, Betu has been reported anti‐tumor effects in lung cancer cells [20]. Furthermore, eEF2K hyperactivation is found in pulmonary arterial hypertension (PAH), a devastating cardiovascular disease, where pharmacological inhibition of eEF2K exhibits therapeutic efficacy in PAH [55]. Our findings demonstrate that Betu mitigates AD‐related cognitive deficits by suppressing eEF2K activity, suggesting its potential therapeutic effect in other diseases associated with eEF2K hyperactivation.

Despite the promising results, several limitations should be acknowledged. The in vivo experiments were conducted using an Aβ_42_‐induced mouse model, which does not fully capture the multifactorial pathology of AD, including tau pathology. Future studies using transgenic mice with Aβ and tau pathology will be necessary to provide a more comprehensive understanding of Betu's effect and mechanisms. Additionally, a more detailed dose–response analysis with a broader gradient of doses is needed to define the optimal therapeutic window. Addressing these limitations will be essential to advance Betu as a therapeutic candidate for AD and to enable its future clinical development.

## 5 Conclusions

This study demonstrates that Betu confers neuroprotective effects against Aβ_42_‐induced neurotoxicity by modulating the eEF2K/eEF2 pathway. Betu restored protein synthesis, preserved dendritic spine integrity, and reduced oxidative stress in hippocampal neurons. In an Aβ_42_‐induced mouse AD model, Betu improved the cognitive decline, accompanied by attenuated neuropathological damage and decreased eEF2 phosphorylation in the hippocampus. Collectively, these findings support Betu as a promising lead compound for AD treatment via the regulation of the eEF2K/eEF2 pathway. Future studies are warranted to evaluate the pharmacokinetics and blood–brain barrier penetration of Betu, which represent critical steps toward clinical translation.

## Author Contributions

L.S., S.Z., D.L. and N.M. designed the study. C.W., X.H., Y.L., Y.P., Q.M., Y.W., and S.‐Q.W. performed the experiments. C.W., X.H., and Y.L. analyzed raw data. Y.P. and Q.M. carefully reviewed the data. Z.Z., L.W., Z.‐R.T., Y.P., K.K.‐L.Y., N.M. and D.L. provided resources. L.S. and S.Z. provided funding support. C.W. and X.H. wrote the original manuscript. L.S., S.Z., D.L. and N.M. reviewed and edited the manuscript. All authors read and approved the final manuscript.

## Funding

This work was supported by the National Key Research and Development Program of China, 2022YFA1104900. The Brain Science and Brain‐like Intelligence Technology‐National Science and Technology Major Project, 2022ZD0214400. National Natural Science Foundation of China, 82371175, U24A20804. Guangdong Basic and Applied Basic Research Foundation, 2023A1515030012, 2022B1515130007, 2023B1515040015. The Science and Technology Projects in Guangzhou, 202102070001. International Science and Technology Projects of Guangdong Province, 2023A0505050121. The Research Project of Guangdong Provincial Bureau of Traditional Chinese Medicine, 20261073.

## Ethics Statement

All animal procedures received approval from the Institutional Animal Care and Use Committee of Guangzhou Ruige Biotechnology (Approval No: 20230417–001).

## Conflicts of Interest

The authors declare no conflicts of interest.

## Supporting information


**Figure S1:** Betu shows no significant effect on the phosphorylation of eIF2α and eIF4B. (A) Representative Western blots depicting p‐eIF2α, eIF2α, and β‐actin expression levels in HT22 cells after treatment with Aβ_42_ (1 μM) and Betu (10, 20, 40 μM) for 24 h. (B) Quantitative analysis of p‐eIF2α/eIF2α and p‐eIF4B/eIF4B (*n* = 3). One‐way ANOVA followed by Dunnett's multiple comparisons test. (C) Representative Western blots depicting p‐eIF4B, eIF4B, and β‐actin expression levels in HT22 cells after treatment with Aβ_42_ (1 μM) and Betu (10, 20, 40 μM) for 24 h. (D) Quantitative analysis of p‐eIF4B/eIF4B (*n* = 3). One‐way ANOVA followed by Dunnett's multiple comparisons test. Data are presented as mean ± SEM.
**Figure S2:** Quantitative analysis of β‐actin in Cellular thermal shift assay (CETSA). CETSA curves of β‐actin in HT22 cells were determined in the absence and presence of Betu. Intensity of protein was normalized with respect to that obtained at 51°C (*n* = 4).
**Figure S3:** Pathological staining and serum biochemistry of Aβ42‐induced mice after treatment with Betu. (A) Body weight of mice. Two‐way ANOVA followed by Dunnett's multiple comparisons test. (B) Hematoxylin and eosin (H&E) staining photomicrographs of mice heart, liver, lung, kidney, and colon. Scale bar: 100 μm. (C–H) Serum biochemical analysis of mice: alanine aminotransferase (ALT) (C), aspartate aminotransferase (AST) (D), creatinine (CREA) (E), uric acid (UA) (F), triglycerides (TG) (G), and total cholesterol (CHO) (H). Group sizes: Ctrl (*n* = 10), Aβ + model (*n* = 12), Aβ + Betu‐25 (*n* = 11), Aβ + Betu‐100 (*n* = 13), Aβ + DNP (*n* = 12). *p < 0.05, **p < 0.01, ****p < 0.0001. One‐way ANOVA followed by Dunnett's multiple comparisons test. ALL data are represented as mean ± SEM.

## Data Availability

The data that support the findings of this study are available on request from the corresponding author. The data are not publicly available due to privacy or ethical restrictions.
