# Supplementary material for: Betulinaldehyde Ameliorates Aβ‐Induced Neurotoxicity and Cognitive Deficits by Modulating the eEF2K/eEF2 Pathway
Source: CNS Neurosci Ther. 2026 Jul 27;32(7):e71050. doi: 10.1002/cns.71050 (PMC13404250; doi:10.1002/cns.71050)
Supplement: Supplementary file 1 — Figure S1: Betu shows no significant effect on the phosphorylation of eIF2α and eIF4B. (A) Representative Western blots depicting p‐eIF2α, eIF2α, and β‐actin expression levels in HT22 cells after treatment with Aβ42 (1 μM) and Betu (10, 20, 40 μM) for 24 h. (B) Quantitative analysis of p‐eIF2α/eIF2α and p‐eIF4B/eIF4B (n = 3). One‐way ANOVA followed by Dunnett's multiple comparisons test. (C) Representative Western blots depicting p‐eIF4B, eIF4B, and β‐actin expression levels in HT22 cells after treatment with Aβ42 (1 μM) and Betu (10, 20, 40 μM) for 24 h. (D) Quantitative analysis of p‐eIF4B/eIF4B (n = 3). One‐way ANOVA followed by Dunnett's multiple comparisons test. Data are presented as mean ± SEM. Figure S2: Quantitative analysis of β‐actin in Cellular thermal shift assay (CETSA). CETSA curves of β‐actin in HT22 cells were determined in the absence and presence of Betu. Intensity of protein was normalized with respect to that obtained at 51°C (n = 4). Figure S3: Pathological staining and serum biochemistry of Aβ42‐induced mice after treatment with Betu. (A) Body weight of mice. Two‐way ANOVA followed by Dunnett's multiple comparisons test. (B) Hematoxylin and eosin (H&E) staining photomicrographs of mice heart, liver, lung, kidney, and colon. Scale bar: 100 μm. (C–H) Serum biochemical analysis of mice: alanine aminotransferase (ALT) (C), aspartate aminotransferase (AST) (D), creatinine (CREA) (E), uric acid (UA) (F), triglycerides (TG) (G), and total cholesterol (CHO) (H). Group sizes: Ctrl (n = 10), Aβ + model (n = 12), Aβ + Betu‐25 (n = 11), Aβ + Betu‐100 (n = 13), Aβ + DNP (n = 12). *p < 0.05, **p < 0.01, ****p < 0.0001. One‐way ANOVA followed by Dunnett's multiple comparisons test. ALL data are represented as mean ± SEM. [file CNS-32-e71050-s001.docx]

**Supplementary Information**

**Betulinaldehyde ameliorates Aβ-induced neurotoxicity and cognitive deficits by modulating the eEF2K/eEF2 pathway**

Chaoqun Wang^1,2,3,#^, Xiaohe Han^1,2,3,#^, Yali Lin^1,2,3^, Yan Pan^4^, Qiuping Miao^1,2,3^, Yaping Wang^5^, Shu-Qin Wang^1,3^, Zhu Zhang^6^, Luyao Wang^7^, Zhi-Ri Tang^8^, Yinghui Peng^1,2,3^, Ken Kin-Lam Yung^9^, Nan Ma^1,2,3,*^, Dan Lu^5,*^, Shiqing Zhang^1,2,3,*^, Lei Shi^1,2,3,*^

^1^ State Key Laboratory of Bioactive Molecules and Druggability Assessment, Guangdong Basic Research Center of Excellence for Natural Bioactive Molecules and Discovery of Innovative Drugs, Jinan University, Guangzhou 510632, China

^2^ JNU-HKUST Joint Laboratory for Neuroscience and Innovative Drug Research, College of Pharmacy, Jinan University, Guangzhou 510632, China

^3^ Guangdong Province Key Laboratory of Pharmacodymamic Constituents of TCM & New Drugs Research, Guangdong Hong Kong-Macau Joint Laboratory for Pharmacodynamic Constituents of TCM and New Drugs Research, Jinan University, Guangzhou 510632, China

^4^ Department of Biology, Hong Kong Baptist University, Kowloon Tong, Kowloon, Hong Kong SAR, China

^5^ Department of Neurology and Stroke Center, The First Affiliated Hospital of Jinan University, Guangzhou 510632, China

^6^ Teaching and Research Division, School of Chinese Medicine, Hong Kong Baptist University, Kowloon Tong, Kowloon, Hong Kong SAR, China

^7^ Law Sau Fai Institute for Advancing Translational Medicine in Bone & Joint Diseases (TMBJ), Hong Kong Baptist University, Kowloon Tong, Kowloon, Hong Kong SAR, China

^8^ School of Intelligent Systems Science and Engineering, Jinan University, Guangzhou 510632, China

^9^ Department of Science and Environmental Studies, The Education University of Hong Kong, Tai Po, Hong Kong SAR, China

# These authors contributed equally to this study.

* Corresponding authors:

Nan Ma, Ph.D. (State Key Laboratory of Bioactive Molecules and Druggability Assessment, Jinan University, Guangzhou 510632, China. E-mail: nanma927@126.com. Tel.: +86-020-85220610)

Dan Lu, Ph.D. (Department of Neurology and Stroke Center, The First Affiliated Hospital of Jinan University, Guangzhou 510632, China. E-mail: ludan@jnu.edu.cn. Tel.: +86-020-85227821)

Shiqing Zhang, Ph.D. (State Key Laboratory of Bioactive Molecules and Druggability Assessment, Jinan University, Guangzhou 510632, China. E-mail: sqzhang@jnu.edu.cn. Tel.: +86-020-85227821)

Lei Shi, Ph.D. (State Key Laboratory of Bioactive Molecules and Druggability Assessment, Jinan University, Guangzhou 510632, China. E-mail: t_shilei@jnu.edu.cn or sophielshi80@gmail.com. Tel.: +86-020-85227821)

**Supplementary methods**

**Hematoxylin and eosin (H&E) staining**

H&E staining was performed utilizing a H&E stain kit (G1076, Servicebio, China). Tissue sections were first submerged in hematoxylin solution for 5 minutes, followed by rinsing with tap water. Subsequently, the sections were briefly immersed in 95% ethanol for 1 minute and stained with eosin dye for 15 seconds. Finally, the sections were dehydrated, sealed, and imaged using a digital slide scanner (Pannoramic MIDI, 3DHISTECH Ltd, Hungary).

**Serum biochemistry analysis**

Blood samples were collected and centrifuged at 3,000 rpm for 15 minutes to separate serum. Serum chemistry analysis was performed to quantify the levels of alanine aminotransferase (ALT), aspartate aminotransferase (AST), uric acid (UA), triglycerides (TG), creatinine (CREA), and total cholesterol (CHO), using a Chemray 800 Auto Chemistry Analyzer (Rayto Life and Analytical Sciences Co., China).

**Supplementary figures**


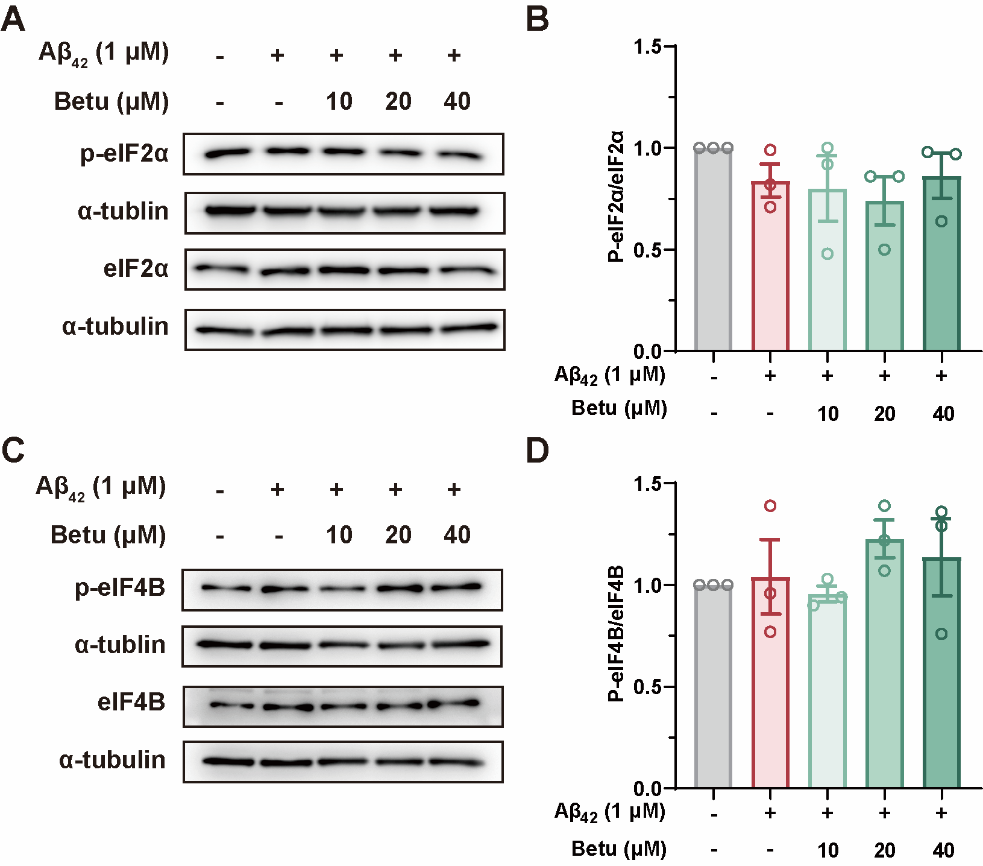


**Fig. S1. Betu shows no significant effect** **on the phosphorylation of eIF2α and eIF4B.** (A) Representative Western blots depicting p-eIF2α, eIF2α, and β-actin expression levels in HT22 cells after treatment with Aβ_42_ (1 μM) and Betu (10, 20, 40 μM) for 24 hours. (B) Quantitative analysis of p-eIF2α/eIF2α and p-eIF4B/eIF4B (*n* = 3). One-way ANOVA followed by Dunnett's multiple comparisons test. (C) Representative Western blots depicting p-eIF4B, eIF4B, and β-actin expression levels in HT22 cells after treatment with Aβ_42_ (1 μM) and Betu (10, 20, 40 μM) for 24 hours. (D) Quantitative analysis of p-eIF4B/eIF4B (*n* = 3). One-way ANOVA followed by Dunnett's multiple comparisons test. Data are presented as mean ± SEM.


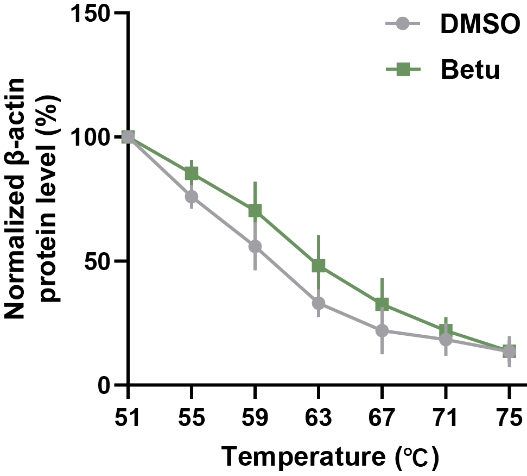


**Fig. S2. Quantitative analysis of β-actin in Cellular thermal shift assay (CETSA).**

CETSA curves of β-actin in HT22 cells were determined in the absence and presence of Betu. Intensity of protein was normalized with respect to that obtained at 51°C (*n* = 4).


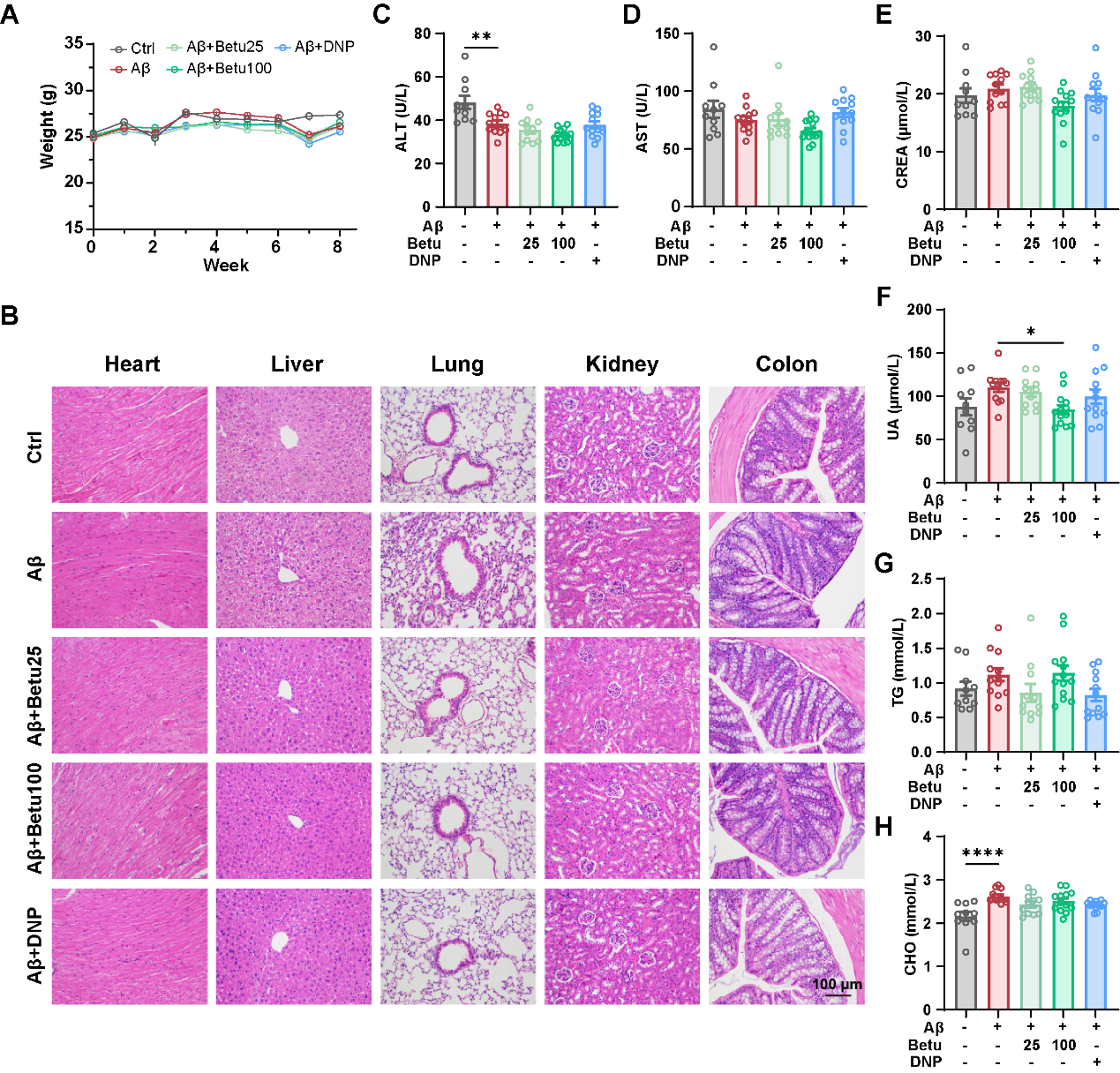


**Fig. S3. Pathological staining and** **serum biochemistry of Aβ_42_-induced mice after treatment with Betu.** (A) Body weight of mice. Two-way ANOVA followed by Dunnett's multiple comparisons test. (B) Hematoxylin and eosin (H&E) staining photomicrographs of mice heart, liver, lung, kidney, and colon. Scale bar: 100 μm. (C–H) Serum biochemical analysis of mice: alanine aminotransferase (ALT) (C), aspartate aminotransferase (AST) (D), creatinine (CREA) (E), uric acid (UA) (F), triglycerides (TG) (G), and total cholesterol (CHO) (H). Group sizes: Ctrl (*n* = 10), Aβ+model (*n* = 12), Aβ+Betu-25 (*n* = 11), Aβ+Betu-100 (*n* = 13), Aβ+DNP (*n* = 12). **P* < 0.05, ***P*<0.01, *****P* < 0.0001. One-way ANOVA followed by Dunnett's multiple comparisons test. ALL data are represented as mean ± SEM.
